# Delineation of Inheritance Pattern of Aleurone Layer Colour Through Chemical Tests in Rice

**DOI:** 10.1186/s12284-017-0187-9

**Published:** 2017-11-21

**Authors:** Chandu Singh, Sripathy K.V., Jeevan Kumar S.P., Bhojaraja Naik K., Govind Pal, Udaya Bhaskar K., Ramesh K.V., Somasundaram G.

**Affiliations:** 1 ICAR- Indian Institute of Seed Science, Mau, Uttar Pradesh 275103 India; 2 Seed Production Unit, ICAR-Indian Agricultural Research Institute, New Delhi, 110012 India

**Keywords:** Aleurone layer, Alleles, Complementary gene action, Duplicate gene action, Rice

## Abstract

**Background:**

Rice aleurone layer develops different colours with various chemical tests that may help to develop some rapid tests for identification/grouping of rice varieties. Understanding the colour inheritance pattern could enable to develop chemical clues that may help for genetic purity analysis along with grow-out-test.

**Results:**

In this study, inheritance pattern of aleurone layer colour was studied in parents, F_1_ and F_2_ progenies derived from the crosses IR 36 **×** Acc. No. 2693 and IR 64 **×** Acc. No. 2693. The parent IR 36 showed light yellow (NaOH/KOH) and brown (phenol/modified phenol test) colour; whereas, Acc. No. 2693 revealed wine red/dark wine red (NaOH/KOH) and light brown colour/no reaction (phenol/modified phenol test). In contrary, another parent IR 64 exhibited light yellow (KOH/NaOH) and dark brown (phenol, modified phenol) colour. Both the F_1_ showed an intermediate light wine red colour (NaOH/KOH) and dark brown (phenol and modified phenol) colour, which is dominant over their one of the parents. The colour pattern with standard phenol/modified phenol, NaOH and KOH tests in F_2_ progenies of both the crosses showed 9:7 (complementary gene interaction) and 11:5 ratios (reciprocal dominance modification of recessive alleles), respectively.

**Conclusions:**

Our findings clearly elucidate the colour inheritance pattern in rice that may facilitate to develop rapid chemical tests to identify/ group the varieties for genetic purity analysis.

## Background

Chemical tests in rice, so far have been extrapolated for identification/characterization of cultivars on the basis of colour pattern in aleurone layer that might developed through enzyme mediated reactions. Aleurone layer is a living entity, which constitute outermost layer of endosperm, specialised in de novo synthesis of reserve mobilizing enzyme complex during seed germination process (Kumar et al. 2015). In addition, the aleurone layer is also involved in the synthesis of oxidase enzymes such as laccase, tyrosinase, polyphenol oxidase, monophenol oxidase and horse-radish peroxidase, which catalyzes to form a colour reaction (Cabaj et al. 2010; Fernandes et al. 2005). Among these oxidases, polyphenol oxidase (PPO) is one of the enzyme that is involved in oxidation of phenol colour reaction through formation of brown coloured melanin pigment (Steffens et al. 1994; Kumar et al. 2016). Polyphenol oxidases avail molecular oxygen, which undergoes hydroxylation and dehydrogenation of phenolic compounds to form reactive *o-*quinones. These *o-*quinones alkylate nucleophilic groups and self-polymerize to form melanin polymers (Fuerst et al. 2014). Role of oxidases is reported to be multifaceted, wherein they are involved in potential seed defense pathways and located in aleurone layer as indicated by their increased levels in the aleuronic fraction (Fraignier et al. 2000; Kumar et al. 2017a; Sinha et al. 2016). Proteomic analysis of aleurone layer in wheat suggested the presence of oxidases along with proteins involved in metabolism (Jerkovic et al. 2010; Kumar et al. 2017b).

In rice, genetic studies on inheritance of colour formation in aleurone layer have been limited. However, biochemical aspects of various enzymes secreted through aleurone layer especially PPO and other reserve mobilizing enzymes have been well studied. Many workers (Joshi and Banerjee 1970; Joshi and Banerjee 1969; Jimenez and Dubcovsky 1999) studied the colour formation of wheat polyploids that had developed colour when reacted with phenol and tyrosine substrates mediated by PPO. Further, reactions of whole-wheat seeds with phenol (Joshi et al. 1969: Maguire et al. 1975) and catechol (Milner and Gould 1951) have been used for cultivar purity testing. Miczynski (1938) reported the presence of one or two genes in bread wheat, which controlled the phenol colour reaction. Moreover, various chemical tests were used so far in identification and differentiation of crop varieties based on the colour differences generated in the aleurone layer when reacted with different chemical tests. Besides, genetic background of the varieties also plays an important role in the identification of varieties. The colour formation by enzyme system has been reported in pearlmillet (Varier et al. 1995), foxtail millet (Pallares et al. 2004), sorghum (Thangavel et al. 2005), rice (Mor et al. 2006; Dileepkumar et al., 2015; Chandu et al. 2017) and wheat (Joshi et al. 2007), respectively. Chemical tests such as FeSO_4_ (Pallares et al. 2004), KOH (Mor et al. 2006), phenol and modified phenol tests (Joshi et al. 2007; Banerjee and Chandra 1977) etc. were studied for the development of seed keys. The purpose of the present study is to determine the inheritance pattern and segregation of colour formation trait in aleurone layer of rice using parents, F_1_ and F_2_ progenies derived from the crosses IR 36 **×** Acc. No. 2693 and IR 64 **×** Acc. No. 2693 by chemical tests.

## Results and Discussion

### Aleurone Layer Colouration in Parents IR 36 × Acc. No. 2693 and IR 64 × Acc. No. 2693

**Fig. 1:**
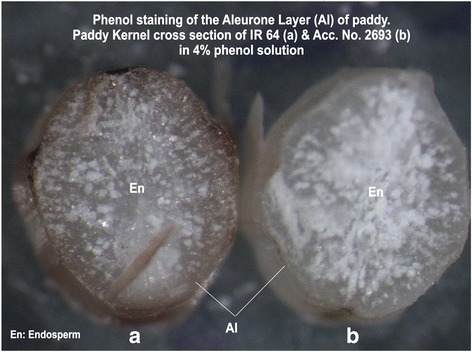
Cross section of rice kernel derived from IR 64 (coloured) and Acc. No. 2693 after subjection to phenol test. Phenol staining of the Aleurone Layer (AL) of paddy. Paddy Kernel cross section of IR 64 (**a**) & Acc. No. 2693 (**b**) in 4% phenol solution

Studies on colour formation of aleurone layer revealed that IR 36 showed light yellow with NaOH and KOH tests, whereas, phenol and modified phenol tests recorded brown colour. The Acc. No. 2693 recorded wine red / dark wine red colour with NaOH, KOH and light brown colour/ no reaction colour with phenol and modified phenol tests, respectively. Similarly, IR 64 recorded light yellow colour with NaOH and KOH tests; while phenol and modified phenol tests were recorded dark brown colour as shown in Table 1. Colour formation with phenol test is depicted in Fig. 1, where the genotypes were grouped based on the biochemical tests and were in congruence with other studies (Thangavel et al. 2005; Nethra et al. 2007; Vijayalakshmi and Vijay 2009; Singh et al. 2011; Anitalakshmi et al. 2014; Kumar et al. 2015).

**Table 1:** Aleurone layer color reaction of parents and F_1_ to different chemical tests

Chemical tests	IR 36	IR 64	Acc. No. 2693	F_1_
NaOH	LY	LY	WR	LWR
KOH	LY	LY	DWR	LWR
Phenol	B	DB	LB/NC	DB
Modified Phenol	B	DB	LB/NC	DB

*LY* Light Yellow, *WR* Wine Red, *DWR* Dark Wine Red, *LWR* light wine red, *LB* Light Brown, *B* Brown, *DB* Dark Brown, *NC* No reaction

### Aleurone Layer Colour Inheritance in F_1_ Plant of IR 36 × Acc. No. 2693 and IR 64 × Acc. No. 2693

**Fig. 2:**
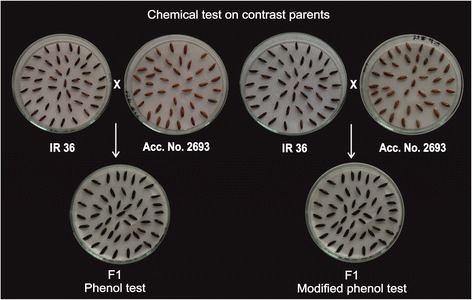
Colour formation in aleurone layer of parents IR 36 (brown colour), Acc. No. 2693 (light brown/no reaction) and their F_1_ progeny (brown colour) with phenol and modified phenol tests

The F_1_s were derived from the crosses IR 36 × Acc. No. 2693 and IR64 × Acc. No. 2693, respectively (Table 1). The freshly harvested F_1_ seeds (derived from cross IR 36 × Acc. No. 2693, as depicted in Fig. 2) showed light wine red colour with NaOH and KOH tests; while brown colour was recorded with phenol and modified phenol tests, respectively.

**Fig. 3:**
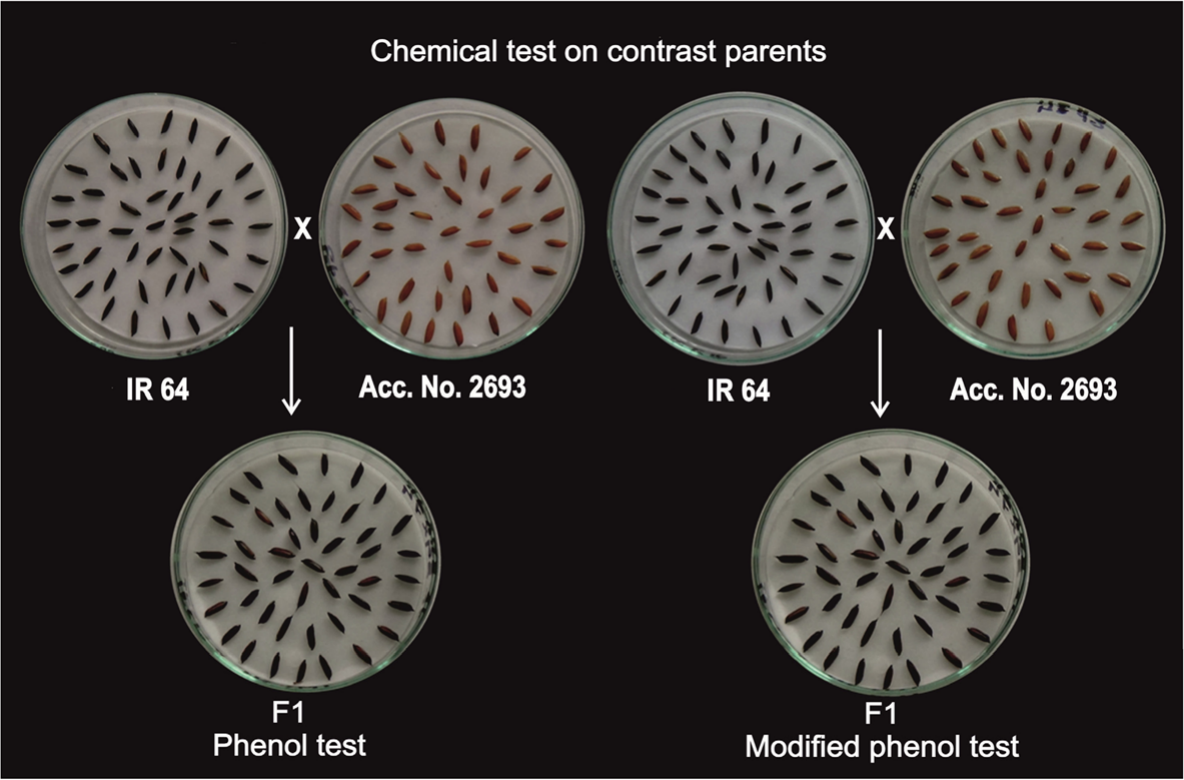
Colour formation in aleurone layer of parents IR 64 (dark brown colour), Acc. No. 2693 (light brown/No reaction) and their F_1_ progeny (dark brown colour) with phenol and modified phenol tests

Further, the freshly harvested F_1_ seeds derived from the cross IR 64 × Acc. No. 2693 (Fig. 3) showed light wine red colour with NaOH and KOH tests, respectively. In contrary, reaction with phenol and modified phenol tests showed dark brown colour, respectively. NaOH and KOH tests showed intermediate colour segregation i.e. light wine red colour compared with parents. In the same way, in case of phenol and modified phenol tests showed dark brown colour from cross IR 64 × Acc. No. 2693 and the similar colour pattern was recorded in case of IR 36 × Acc. No. 2693 that implies that the observed colour is dominant over light yellow.

### Aleurone Layer Colour Inheritance in F_2_ Progenies Derived from IR 36 × Acc. No. 2693 and IR 64 × Acc. No. 2693 with Standard Phenol and Modified Phenol (copper sulphate) Tests

Out of 484 F_2_ progenies of cross IR 36 × Acc. No. 2693; 273 and 255 F_2_ progenies were brown/dark brown in colour, whereas 211 and 229 F_2_ progenies showed light brown/no colour with standard phenol and modified phenol tests, respectively. Further, 420 F_2_ progenies derived from cross IR 64 × Acc. No. 2693 were evaluated. It is observed that 251 and 246 F_2_ progenies were brown/dark brown in colour; 169 and 174 F_2_ progenies showed light brown/no reaction with standard phenol and modified phenol tests, respectively. Therefore, the investigation revealed that the colour trait in the aleurone layer of F_2_ progenies were segregated with complementary gene interaction with a ratio of 9:7 indicates a goodness of fit with observed ratio (Table 2). The F_2_ colour segregation was consistent with the complementary gene interaction (9:7) for all F_2_ progenies of both the crosses. Therefore, two major genes and their alleles with complementary gene action controls the colour formation in aleurone layer.

**Table 2:** Aleurone layer color segregation in F_2_ progenies of the crosses IR 36 × Acc.No. 2693 and IR 64 × Acc. No. 2693for standard phenol and modified phenol with copper sulphate tests

Cross	Chemical tests	Class	Brown/dark brown colour	Light brown/ No reaction	Total	Ratio	χ2	*P* value (at 1 degrees of freedom)
IR 36 × Acc. No. 2693	Phenol test	Observed	273.00	211.00	484	9:7	0.005	0.945
		Expected	272.25	211.75				
	Modified phenol test	Observed	255.00	229.00	484	9:7	2.498	0.114
		Expected	272.25	211.75				
IR 64 × Acc. No. 2693	Phenol test	Observed	251.00	169.00	420	9:7	2.104	0.147
		Expected	236.25	183.75				
	Modified phenol test	Observed	246.00	174.00	420	9:7	0.920	0.338
		Expected	236.25	183.75				

**Fig. 4:**
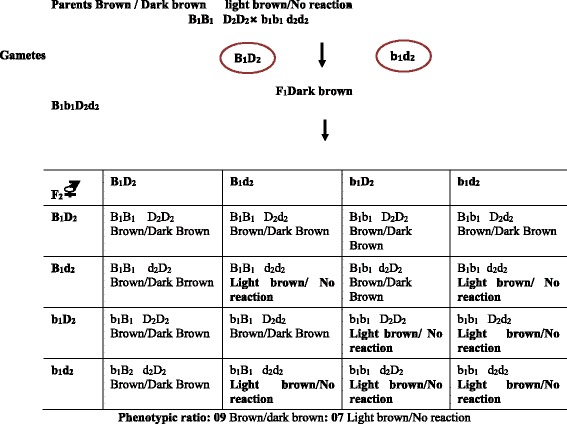
Complementary gene interaction in the development of aleurone layer colour through standard phenol and modified phenol tests in rice seed giving rise to the phenotypic ratio of 9:7 (brown/dark brown: light brown/no reaction) in F_2_ progenies

The development of colour in aleurone layer (brown/ dark brown) with standard phenol and modified phenol tests (copper sulphate) in rice requires the presence of two dominant genes, B_1_ and D_2_ e.g. B_1_B_1_ D_2_D_2_ (Fig. 4). When either B_1_ (e.g. b_1_b_1_ D_2_D_2_) or D_2_ (B_1_B_1_ d_2_d_2_) or both the genes (e.g. b_1_b_1_ d_2_d_2_) are present in homozygous recessive condition, brown/ dark brown colour cannot be produced; as a result, light brown / no reactions were obtained. The brown/dark brown colour variety (IR 36 and IR 64) of rice (B_1_B_1_ D_2_D_2_) was crossed to a light brown/no reaction variety (Acc. No 2693) with the genotype b_1_b_1_ d_2_d_2_ showed dark brown colour in the F_1_ (B_1_b_1_ D_2_d_2_) progeny. In the F_2_ progenies, on an average nine progenies had one dominant allele of both the genes B_1_ and D_2_. These plants, therefore has brown/ dark brown colour. Three, out of sixteen F_2_ progenies, shall have dominant B_1_ but had homozygous recessive genotype b_1_b_1_; three others shall have dominant D_2_ but had homozygous recessive genotype d_2_d_2_, while one plant had both the genes in homozygous recessive genotype b_1_b_1_ d_2_d_2_. All these (seven progenies) had light brown/no reaction. In this type of gene interaction, the production of one of the two phenotypes of colour trait requires the presence of dominant alleles of both the genes controlling the concerned trait. When any one of the two or both the genes are present in the homozygous recessive state, the contrasting phenotype is produced, which leads to various modifications of the typical dihybrid, trihybrid etc. F_2_ ratio.

### The Mechanism of Colour Formation in Aleurone Layer

**Fig. 5:**
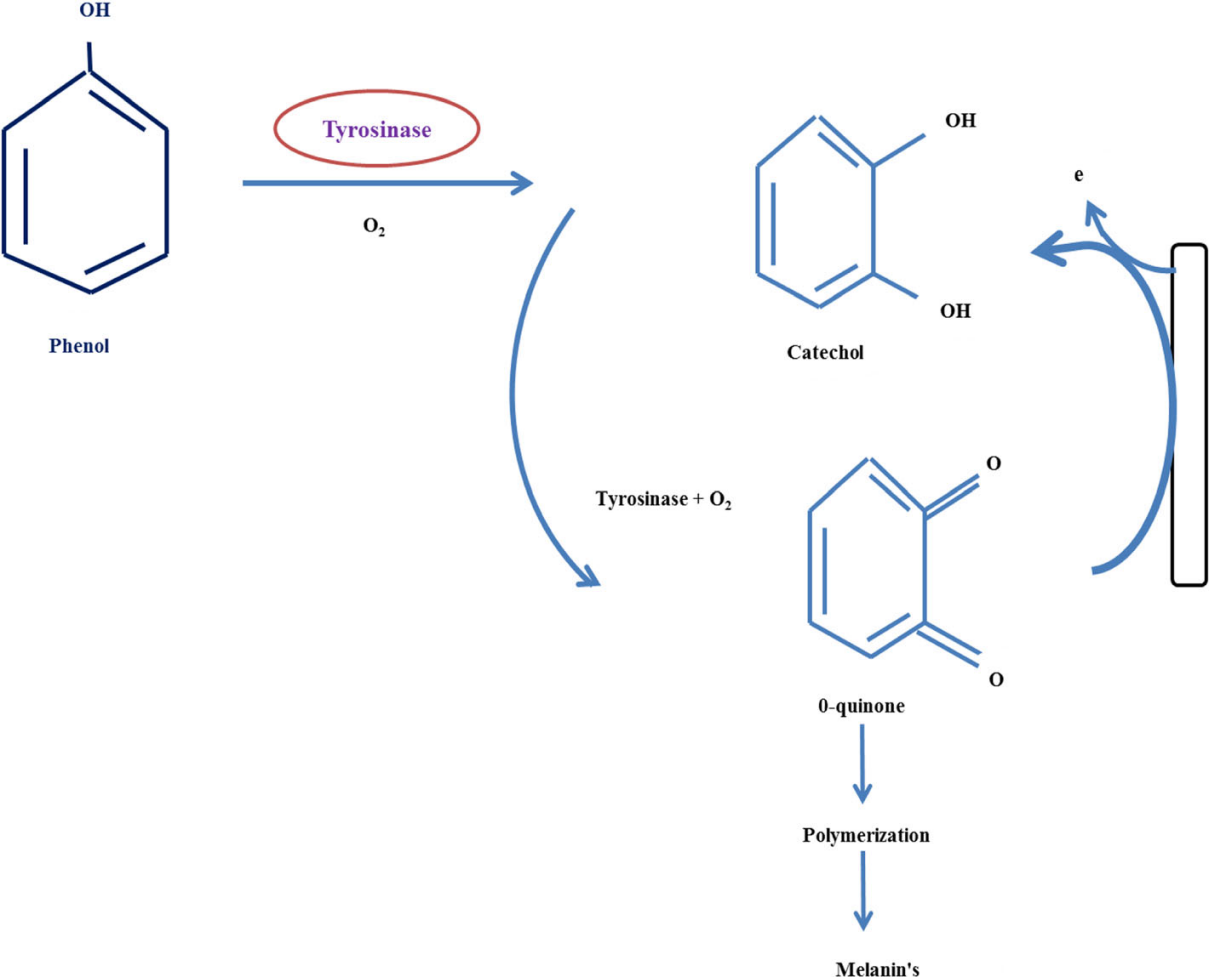
Mechanism of melanin colour formation in seed aleurone layer using enzyme system upon reaction with phenol test

Phenol test, which is an index of polyphenol oxidase activity, has been reported to be associated with intra-varietal diversity that has been used in ascertaining varietal purity. Colour formation in aleurone layer occurs by phenol oxidation in two reactions (Fig. 5). In the first reaction, the aromatic ring of phenol can be hydroxylated to form catechols or quinols, respectively. In the second reaction, the quinols or catechols undergo oxidation to form quinones (Takahashi, 1984). Two major genes and their allelic interactions control this reaction, which is localized in seed aleurone layer in rice. The ability of genotype to form colour depends on the tyrosinase activity, which is located at aleuronic layer (Masuthi et al. 2015). The extent of colour intensity among germplasms varied because of differences in enzyme activity, temperature, light, aeration and genetic background, respectively (Sivasubramanian and Ramakrishnan 1974). On the other hand, the germplasms with no colour might have resulted because of inability to hydroxylate the aromatic ring of phenol either due to shortage of electron donor or hydroxylating enzyme (Takahashi and Hamza 1983).

The results of phenol test are usually distinct and easily interpreted. Walls (1965), reported that the phenol colour reaction depends on the quality and quantity of oxidases present in the seeds, whereas Takahashi and Hamza (1983), reported that monophenol oxidase was extremely localized in aleurone layer of grains even though it is present in all other plant parts of a variety. Presence of enzyme system has been utilized to distinguish the crop varieties in rice and tomato (Pieper 1922; Joshi and Banerjee 1970; Mansing 2010; Vijayalakshmi and Vijay 2009; Anitalakshmi et al. 2014; Sripunitha and Sivasubramaniam 2014; Vishwanath et al. 2013).

Qian et al. (2000) reported major QTL (qPH-4a) that is responsible for phenol colour has been located on chromosome 4, which explained the total phenotypic variation as high as 94.6%. Hence, this QTL is controlled by major gene. Moreover, two minor QTLs (qPH-1 and qPH-4b) located on chromosome 1 and 4 have account of total phenotypic variation of 14.9 and 29.5%, respectively. Hence, minor genes that code for these QTLs, which control phenol colour have showed positive additive effects. Phenol showed a bimodal distribution in the double haploid population and a major gene detected was close to the *Ph* gene located on chromosome 4 (Lin et al. 1994).

### Aleurone Layer Colour Inheritance in F_2_ Population Derived from IR 36 × Acc. No. 2693 and IR 64 × Acc. No. 2693 with Sodium Hydroxide (NaOH) and Potassium Hydroxide (KOH) Tests

The 484 F_2_ progenies of the cross IR 36 × Acc. No. 2693 were evaluated. Among them, 319 and 335 F_2_ progenies were wine red/dark wine red colour, whereas 165 and 149 F_2_ progenies were light yellow coloured with NaOH and KOH tests, respectively. Further, 420 F_2_ progenies derived from the cross IR 64 × Acc. No. 2693 were evaluated, of which 281 and 290 F_2_ progenies were wine red/dark wine red colour and 139 and 130 F_2_ progenies were light yellow coloured with NaOH and KOH tests, respectively.

The segregation of aleurone layer colour in 11:5 ratio for wine red/dark wine red and light yellow in F_2_ progenies of both the crosses showed that the colour trait is governed by two genes, where a dominance of one gene is modified by homozygous recessive condition of another gene. The two genes that interact to produce a single character may also reciprocally modify the dominance relationship between the alleles at the other locus. Thus, the typical 15:1 ratio for duplicate gene action is modified as 11:5 due to the reciprocal dominance modification of recessive alleles. This indicates a goodness of fit with expected ratio of 11:5 for the reciprocal dominance of duplicate genes as given in Table 3.

**Table 3:** Aleurone layer color segregation in F_2_ progenies of the crosses IR 36 × Acc.No. 2693 and IR 64 × Acc. No. 2693 for NaOH and KOH tests

Cross	Chemical tests	Class	Wine red/dark wine red	Light yellow/ No reaction	Total	Ratio	χ2	P value(at 1 degrees of freedom)
IR 36 × Acc. No. 2693	NaOH	Observed	319.00	165.00	484	11:5	1.818	0.178
		Expected	332.75	151.25				
	KOH	Observed	335.00	149.00	484	11:5	0.048	0.825
		Expected	332.75	151.25				
IR 64 × Acc. No. 2693	NaOH	Observed	281.00	139.00	420	11:5	0.665	0.415
		Expected	288.75	131.25				
	KOH	Observed	290.00	130.00	420	11:5	0.017	0.895
		Expected	288.75	131.25				

**Fig. 6:**
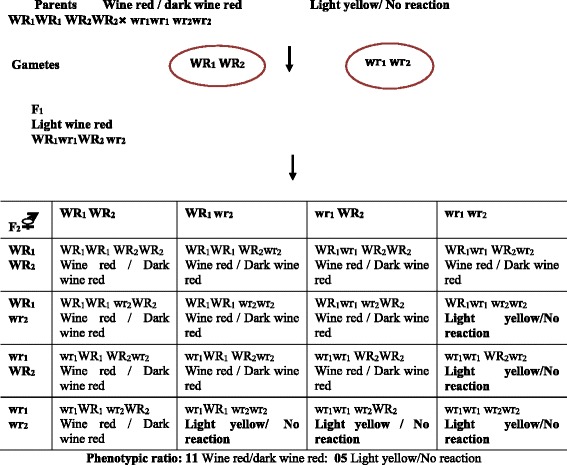
Dominance modification of duplicate genes leading to a 11:5 phenotypic ratio in the F_2_ progenies for the presence of wine red / dark wine red and light yellow/no reaction colouration of aleurone layer of rice seed with NaOH and KOH tests

Two genes, WR_1_ and WR_2_ showing duplicate interaction governs the development of aleurone layer colour (wine red / dark wine red/light) with NaOH and KOH in rice plants. However, the recessive homozygous condition of one gene, say wr_1_ wr_1_ reverses the dominance relationship at the other locus; hence, the genotype wr_1_wr_1_ WR_2_ wr_2_ produces the same phenotype as the homozygous double recessive wr_1_ wr_1_, wr_2_ wr_2_ of light yellow/no reaction._._ Similarly, homozygous recessive condition of the other gene, wr_2_ wr_2_ has the same effect on the dominance relationship at the wr_1_ locus. As a consequence, the genotype WR_1_ wr_1_ wr_2_ wr_2_ produces the same phenotype as the double recessive homozygote wr_1_ wr_1_ wr_2_ wr_2_ of light yellow/no reaction. Therefore, the genotypes WR_1_ wr_1_ wr_2_ wr_2_; wr_1_ wr_1_ WR_2_ wr_2_ and wr_1_ wr_1_ wr_1_ wr_2_ all produce light brown/no reaction condition. Thus, the dominant genes WR_1_ and WR_2_ behave as if, they were recessive to their allele’s wr_1_ and wr_2,_ respectively; whenever they are present in association with the homozygous recessive state at the other locus that is with wr_2_ wr_2_ and wr_1_ wr_1,_ respectively as depicted in Fig. 6.

The wine red/ dark wine red colour variety (Acc. No. 2693) of rice (WR_1_WR_1_ WR_2_ WR_2_) was crossed with a light yellow/no reaction variety (IR 36 and IR 64) with the genotype wr_1_wr_1_, wr_2_wr_2_, the derived F_1_ (WR_1_ wr_1_ WR_2_ wr_2_) has produced light wine red colour (intermediate) even in the presence of dominant alleles of both the genes. In the F_2_ generation, on an average nine plants out of 16, have at least one dominant allele of both the genes WR_1_ and WR_2_; these plants develop wine red and dark wine red colour.

One plant has the genotype WR_1_ WR_1_ wr_2_ wr_2,_ while another has the genotype wr_1_ wr_1_ WR_2_ WR_2_. These two plants also develop wine red and dark wine red colour; since, they have either WR_1_ / WR_2_ in the homozygous state, which is able to produce wine red and dark wine red colour. Two plants out of 16 are heterozygous for WR_2_ and homozygous for wr_1_ and vice versa. These four plants do not develop wine red and dark wine red colour; since, the homozygous recessive state of wr_2_ and wr_1_, reverses the dominance relationship between WR_1_/wr_1,_ WR_2_ / wr_2_, respectively. The remaining one plant is also light yellow/no reaction because it is homozygous recessive for both the genes wr_1_ wr_1_ wr_2_ wr_2._ Thus, the typical dihybrid 15:1 ratio for duplicate gene action is modified as 11:5 due to the reciprocal dominance modification of wr_1_ and wr_2._

The present findings were reported in case of cotton (Fuchs et al. 1972). Two genes, G_1_ and G_2_ showing duplicate gene interaction governs the presence of pigment glands on cotton plants. However, the recessive homozygous condition of one gene, say g_1_ g_1,_ reverses the dominance relationship at the other locus so that genotype g_1_ g_1_ G_2_ g_2_ produces the same phenotype as the homozygous double recessive g_1_ g_1_ g_2_ g_2._ Similarly, homozygous recessive condition of the other gene, g_2_ g_2_ has the same effect on the dominance relationship at the g_1_ locus. As a consequence, the genotype G_1_ g_1_ g_2_ g_2_ produces the same phenotype as the double recessive homozygote g_1_ g_1_ g_2_ g_2_ produces glandless plants. Therefore, the genotypes G_1_ g_1_ g_2_ g_2,_ g_1_ g_1_ G_2_ g_2_ and g_1_ g_1_ g_2_ g_2_ all produce glandless condition; hence, the F_2_ phenotypic ratio 11:5 was observed. Similar classification was noticed in the present study with NaOH, which is in congruence with the report in wheat (Mansing 2010), rice (Vanangamudi et al. 1988; Sripunitha and Sivasubramaniam 2014), *urdbean* (Chakrabarthy and Agrawal 1990); cotton (Ponnuswamy et al. 2003; Reddy et al. 2008), safflower (Biradar Patil et al. 2006). In addition, the same findings corroborate with the crops such as sesamum (Suhasini 2006) soybean (Chavan 2010), sunflower (Sathisha et al. 2012; Kallihal et al. 2013) and tomato (Qian et al. 2000), respectively. The reasons for various colour attributed when reacted with sodium hydroxide and potassium hydroxide might be due to inherent chemical difference, stability of genetic characters and secondary metabolites present in the seeds (Masuthi et al. 2015; Vanderburg and Vanzwol 1991; Chakrabarthy and Agrawal 1990).

Therefore, these studies are particularly useful, where non-availability of distinct stable morphological markers for identification of increased varieties. These chemical tests along with other parameters like 1000 seed weight, seed size, response to GA_3_, 2,4-D and soluble proteins acts as a descriptors for identification of the rice varieties. Further, these tests could help to develop a rapid varietal identification that may help the breeders and seed inspectors to monitor the quality seed production (Nethra et al. 2007). Thus, chemical tests are one of the important characters that help in easy identification of varieties for genetic purity.

## Conclusions

Based on the response of biochemical tests with 904 F_2_ progenies derived from crosses, IR 36 × Acc. No. 2693 and IR 64 × Acc. No. 2693 were utilised for delineation of inheritance pattern of aleurone layer colour in rice. In this investigation, it was found that the colour trait in aleurone layer of the F_2_ progenies derived from both the crosses were segregated with complementary gene interaction of 9:7 ratio (brown/dark brown: light brown/no reaction), indicating a goodness of fit with observed ratio for standard phenol and modified phenol tests (CuSO_4_), respectively. Further, the colour trait in F_2_ progenies of both the crosses with NaOH and KOH tests were observed to segregate in 11:5 ratio (wine red/dark wine red: light yellow/ no reaction), wherein typical 15:1 ratio for duplicate gene action is modified as 11:5 due to the reciprocal dominance modification of recessive alleles. Therefore, it is deduced that the colour trait in aleurone layer was found to be controlled by two major genes and their allelic interactions. These findings could be utilised for easy identification of varieties in rice breeding programme, gene expression analysis, cloning and tagging of gene and also to develop the seed keys to precisely define cultivars that would serve an alternative for Grow-out-test.

## Methods

### Plant Materials

The present work was carried out using IR 36, IR 64 and Acc. No. 2693 (as parents), F_1_ and F_2_ progenies. The F_1_s were derived from cross between IR 36 × Acc. No. 2693 and IR 64 **×** Acc. No. 2693, respectively during *kharif* 2014 at ICAR-Directorate of Seed Research (ICAR-DSR), Mau, Uttar Pradesh, India. Both the crosses (F_1_ seeds) were raised during off-season 2014–15 at regional station, ICAR-DSR, GKVK campus, Bengaluru. Further, F_2_ progenies of both the crosses were raised during *kharif* − 2015 at ICAR-DSR, Mau; 484 and 420 F_2_ seeds of both crosses IR 36 × Acc. No. 2693 and IR 64 **×** Acc. No. 2693 were used for present investigation to delineate the inheritance pattern of colour trait in aleurone layer using chemical tests, respectively.

### Chemical Tests

To know the segregation pattern of colour trait in aleuronic layer, study has been performed using different chemical tests such as standard phenol, modified phenol (CuSO_4_), potasium hydroxide and sodium hydroxide tests. These chemical tests are insensitive to environment and serves not only as basis for grouping of varieties, but also used for genotype identification (Naga Padma et al. 1996) with consistent results.

### Phenol Test

For phenol test, seeds were pre-soaked in distilled water for 24 h. Thereafter, they were transferred on two layers of Whatman No.1 filter paper saturated with 4 % phenol solution (Merck, Cat. No. AL8AF58565, Merck Specialities Private Ltd. Mumbai, India). The Petri-dishes were covered and incubated at 25 ± 1 °C and the change in colour of aleurone layer in response to phenol reaction was evaluated after 24 h. The parents, F_1_ and F_2_ progenies were categorized into five categories as no reaction, light brown, brown, dark brown and black colour (Jaiswal and Agrawal 1995).

### Modified Phenol Test-A (CuSO_4_)

Modified phenol test was conducted, which is alike to standard phenol test except that seeds were pre-soaked in 0.5% (*w*/*v*) copper sulphate (Helix Bio-Science, Cat. No.HBC043212, New Delhi, India) solution for 24 h. Colour reaction was noted after 48 h of incubation and the parents; F_1_ and F_2_ progenies were categorized into five categories as no reaction, light brown, brown, dark brown and black colour (Jaiswal and Agrawal 1995).

### Sodium Hydroxide (NaOH) Test

Parents, F_1_ and F_2_ seeds were subjected to sodium hydroxide test where, seeds were pre-soaked in 5 % sodium hydroxide solution (Merck, Cat. No. MJ8D580230, Merck Specialities Private Ltd. Mumbai, India) and kept at room temperature for one hour and change in colour of the solution was observed. Chakrabarty et al. 1989, categorized the reaction into light yellow and wine red based on the intensity of change in colour solution.

### Potassium Hydroxide (KOH) Test

Seeds of parents, F_1_ and F_2_ progenies were pre-soaked in 5 % potassium hydroxide solution (Helix Bio-Science, Cat. No. A3641, New Delhi, India) and kept at room temperature for 4 h and a change in colour of the solution was observed. Based on the intensity of reaction, the populations were categorized into various groups viz.*,* light yellow, dark yellow, light wine red, dark wine red (Vanangamudi et al. 1988).

### Chi-square (χ2) Goodness of Fit Test

Chi-square (**χ**2) goodness of fit test was performed to analyse phenotypic segregation between observed values (O) to the expected values (E) for F_2_ population data, using χ^2^ = ∑(O - E)^2^ /E.

## Abbreviations

Acc. No.: Accession number
B: Brown
b_1_b_1_ d_2_d_2_: Light brown/no reaction
B_1_B_1_: Brown
D_2_D_2_: Dark brown
DB: Dark brown
DWR: Dark wine red
ICAR: Indian Council of Agricultural Research
KOH: Potassium hydroxide
LB: Light brown
LWR: Light wine red
LY: Light yellow
NaOH: Sodium hydroxide
NR: No reaction
WR: Wine red
wr_1_ wr_1_ wr_2_ wr_2_: Light yellow/ no reaction
WR_1_ WR_1_: Wine red
WR_2_ WR_2_: Dark wine red
